# Adult hypophosphatasia with compound heterozygous p.Phe327Leu missense and c.1559delT frameshift mutations in tissue-nonspecific alkaline phosphatase gene: a case report

**DOI:** 10.1186/s13256-019-2045-4

**Published:** 2019-04-24

**Authors:** Kazunori Fukushima, Keiko Kawai-Kowase, Yukio Yonemoto, Makoto Fujiwara, Hiroko Sato, Mahito Sato, Takuo Kubota, Keiichi Ozono, Junich Tamura

**Affiliations:** 1 Department of Emergency Medicine, Gunma University Graduate School of Medicine, Maebashi, Gunma Japan; 2 Department of General Medicine, Gunma University Graduate School of Medicine, 3-39-15 Showa-machi, Maebashi, Gunma 371-8511 Japan; 3 Department of Orthopedic Surgery, Gunma University Graduate School of Medicine, Maebashi, Gunma Japan; 4 Department of Pediatrics, Osaka University Graduate School of Medicine, Suita, Osaka Japan

**Keywords:** Hypophosphatasia, Alkaline phosphatase, Compound heterozygous mutation, Medical checkup, Osteoporosis

## Abstract

**Background:**

Hypophosphatasia is an inherited bone disease characterized by low alkaline phosphatase activity encoded by *ALPL*. Clinically, hypophosphatasia can be categorized as perinatal, infantile, childhood, and adult forms, as well as odonto-hypophosphatasia, according to the age at first sign or dental manifestations. Adult hypophosphatasia typically presents in middle-aged patients who appear to be in good health in early adulthood and manifests as painful feet caused by recurrent, slow-healing stress fractures of the lower limb. Because the symptoms of adult hypophosphatasia vary and are common, many patients with hypophosphatasia might be not diagnosed accurately and thus may receive inappropriate treatment.

**Case presentation:**

We report a case of a 35-year-old Japanese woman with low serum alkaline phosphatase detected at a routine medical checkup. She had mild muscle/bone pain but no history of rickets, fractures, or dental problems. Measurement of bone mineral density of the lumbar spine and the femoral neck revealed osteopenia below the expected range for age in a young adult. Abdominal ultrasonography revealed numerous microcalcifications in both kidneys. Analysis of amino acids in urine revealed that phosphoethanolamine was elevated. Low serum alkaline phosphatase activity, elevation of phosphoethanolamine, and low bone mineral density supported the diagnosis of hypophosphatasia. *ALPL* mutation analysis revealed two mutations: p.Phe327Leu and c.1559delT. These genetic abnormalities were previously reported in perinatal, infantile, and childhood but not adult hypophosphatasia. On the basis of the clinical presentation, laboratory and imaging findings, and genetic analyses, the patient was definitively diagnosed with adult hypophosphatasia. To the best of our knowledge, this is the first case report of adult hypophosphatasia with the compound heterozygous mutations p.Phe327Leu and c.1559delT.

**Conclusions:**

Although the risk of bone fracture was high in this case, treatment approaches differ between osteoporosis and hypophosphatasia. Because adult hypophosphatasia diagnosis is often difficult because of their varied symptoms, hypophosphatasia should be considered in the differential diagnosis of low serum alkaline phosphatase. Early diagnosis is important so that appropriate treatment can be initiated.

## Backgrounds

Low serum alkaline phosphatase (ALP) activity, which is infrequent in adults, can occur in several diseases, such as hypothyroidism, zinc deficiency, and steroid treatment. Hypophosphatasia (HPP) is an inherited systemic disease characterized by low activity of tissue-nonspecific alkaline phosphatase (TNSALP) and defective bone mineralization. Rathbun reported a case of a 2-month-old patient with rickets who had paradoxically low serum ALP activity. Subsequent reports suggested that HPP can result from various mutations in *ALPL* encoding for ALP in liver, bone, or kidney [1, 2]. Currently, more than 320 *ALPL* mutations in patients with HPP have been reported in the *ALPL* gene database [3].

Clinically, HPP can be categorized as perinatal (lethal and benign), infantile, childhood, and adult forms, as well as odonto-hypophosphatasia, according to the age at first sign or dental manifestations [2]. Adult HPP typically presents in middle-aged patients who appear to be in good health in early adulthood and manifests as painful feet caused by recurrent, slow-healing stress fractures of the lower limb [4–7]. Some patients also complain of chronic muscle/bone pain without fractures and reduced muscle strength. Moreover, McKiernan *et al.* suggested that crystalline arthritis, orthopedic surgery, chondrocalcinosis, calcific periarthritis, enthesopathy, and diffuse idiopathic skeletal hyperostosis were more frequent in adult patients with HPP than in the general adult population [8]. Because the symptoms of adult HPP vary greatly and are common in the general population as well, its recognition is challenging, and the possibility remains that many patients with HPP who are not diagnosed accurately might receive inappropriate treatment.

We report a case of a woman with HPP who was referred to our hospital with low serum ALP that was found at an annual medical checkup for workers. Although she had bone and muscle pain but no history of childhood rickets, dental abnormalities, or bone fractures, a genetic analysis was performed, which revealed two *ALPL* mutations (p.Phe327Leu and c.1559delT), which were previously reported in perinatal benign, infantile, and childhood HPP cases [9]. To the best of our knowledge, this is the first case report of adult HPP with the compound heterozygous mutations p.Phe327Leu and c.1559delT.

## Case presentation

A 35-year-old Japanese woman was referred to our hospital for evaluation of low serum ALP at an annual medical checkup for workers. Her serum ALP levels had not been determined before. She did not have symptoms except for mild muscle and bone pain in both lower limbs since childhood, which did not interfere with her daily life. Her physical activity level was normal. She had no history of rickets, fractures, or dental problems. Specifically, she did not have premature loss of her primary dentition, although she had had developmental dysplasia of the hip during infancy. She did not take any medication, including supplements, before admission. She did not smoke and denied alcohol abuse and use of illicit drugs. She had no known allergies. She has been working in the clothing industry for approximately 10 years and living with her parents in a residential area in Japan. Her mother is alive and has had breast cancer, and her older sister had Hashimoto’s thyroiditis. Her father had no major illnesses. Her parents had no history of fractures.

On initial examination, her vital signs were as follows: body temperature, 37.0 °C, blood pressure 115/80 mmHg, pulse 101 beats/min, height 150.3 cm, and body weight 44 kg (body mass index 19.6 kg/m^2^). Examination of the right femur and the left crus revealed spontaneous pain; however, there was no evidence of tenderness or pain with percussion. Examination of palpebral conjunctiva did not suggest anemia, and the bulbar conjunctiva was not icteric. The thyroid was not palpable, and the results of chest and abdominal examinations were normal. The results of neurological examinations, including muscle strength tests, deep tendon reflexes, and esthesia, were also normal except for spontaneous pain in bilateral legs, and no skin lesions were noted.

On initial visit, the patient’s complete blood count was normal (hematocrit, 41.4%; hemoglobin, 13.8 g/dl; red cell count, 4.91 × 10^6^/mm^3^; white cell count, 6600/mm^3^; and platelet count 308 × 10^3^/mm^3^) (Table 1). Laboratory evaluation revealed that serum ALP was remarkably low at 13 U/L, serum iron was low at 40 μg/dl, and serum phosphorus was slightly elevated at 4.3 mg/dl, whereas serum calcium was normal at 10.2 mg/dl. Inflammatory markers (C-reactive protein, 0.02 mg/dl), liver function tests (albumin, 4.9 g/dl; total bilirubin, 0.3 mg/dl; aspartate aminotransferase, 13 U/L; alanine aminotransferase, 6 U/L), renal function tests (blood urea nitrogen, 8 mg/dl; serum creatinine, 0.61 mg/dl), electrolytes (sodium, 139 mEq/L; potassium, 4.3 mEq/L; chloride, 103 mEq/L), thyroid-stimulating hormone (2.35 μU/ml), and free thyroxine (1.59 ng/dl) were within the normal ranges (Table 1).

**Table 1:** Laboratory data on initial visit

Variable	Reference range	On presentation
Hematocrit (%)	35.0–45.0	41.4
Hemoglobin (g/dl)	11.8–15.1	13.8
White cell count (per mm^3^)	4.0–9.6	6.6
Platelet count (per mm^3^)	160,000–350,000	308,000
Red cell count (per mm^3^)	4,000,000–5,000,000	4,910,000
Total protein (g/dl)	6.3–7.9	7.9
Albumin (g/dl)	3.9–5.0	4.9
Total bilirubin (mg/dl)	0.3–1.2	0.3
Aspartate aminotransferase (U/L)	13–33	13
Alanine aminotransferase (U/L)	6–27	6
Lactate dehydrogenase (U/L)	119–229	130
Alkaline phosphatase (U/L)	115–359	13
γ-Glutamyltransferase (U/L)	10–47	9
Uric acid (mg/dl)	2.6–7.0	3.6
Blood urea nitrogen (mg/dl)	8–20	8
Creatinine (mg/dl)	0.46–0.79	0.61
Sodium (mEq/L)	137–145	139
Potassium (mEq/L)	3.5–4.8	4.3
Chloride (mEq/L)	100–107	103
Calcium (mg/dl)	8.9–10.5	10.2
Phosphorus (mg/dl)	2.5–4.1	4.3
Iron (mg/dl)	86–151	40
Zinc (mg/dl)	80-	82.0
IgG (mg/dl)	870–1700	1306
C-reactive protein (mg/dl)	−0.1	0.02
Thyroid-stimulating hormone (mU/ml)	0.390–4.010	2.358
Free thyroxine (ng/dl)	0.83–1.71	1.59

*IgG* Immunoglobulin G

**Fig. 1:**
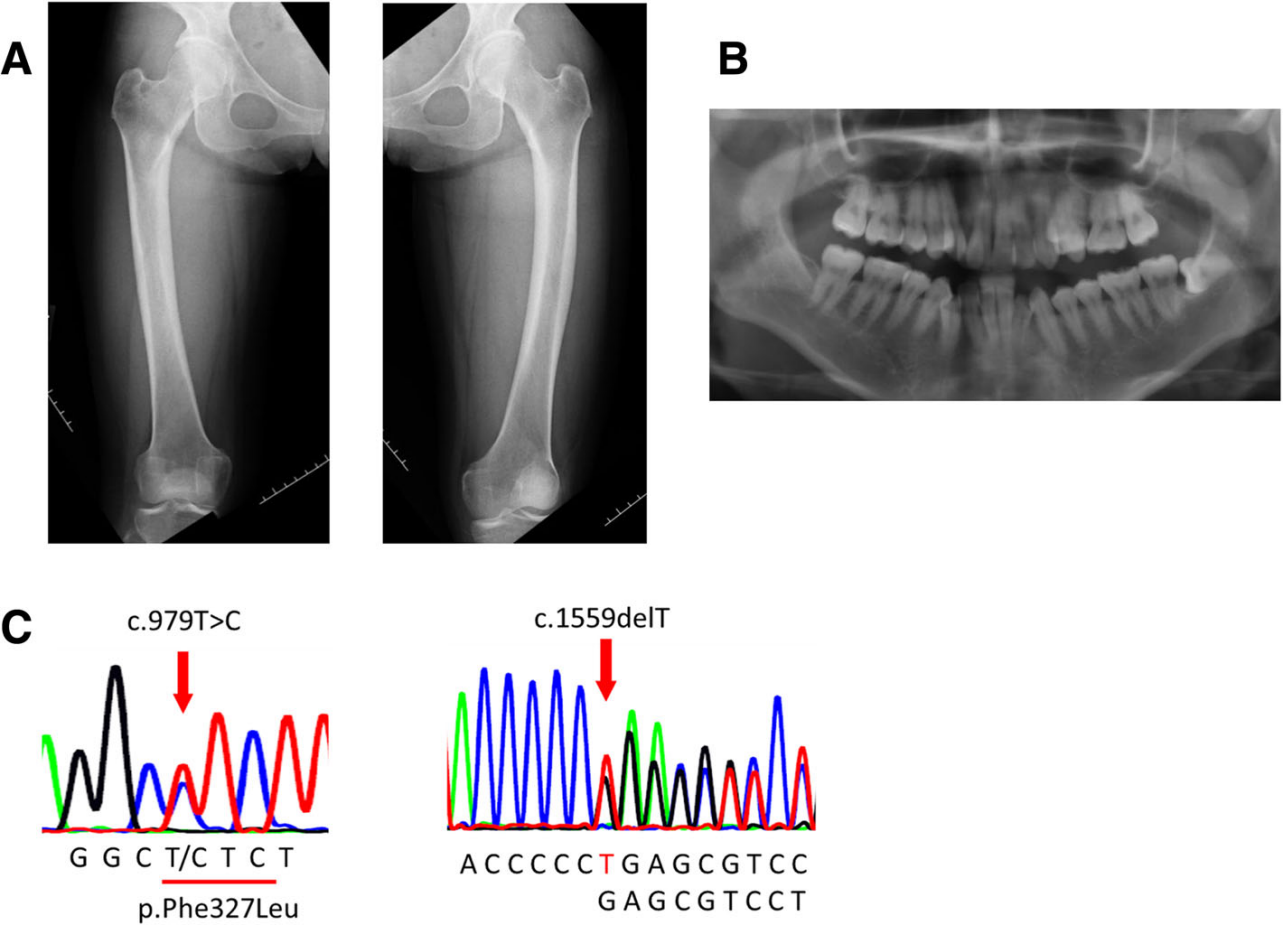
**a** X-ray of lower limbs showing mild lateral bowing in both femurs. **b** Orthopantomography. **c** Sequence of *ALPL* showing nucleotide conversion of T to C in position 979 (*left*) and deletion of T in position 1559 (*right*)

X-rays of the limbs for further evaluation of potential bone abnormalities showed mild lateral bowing of both femurs (Fig. 1a). X-rays of the cervical and lumbar spine showed no scoliosis. Orthopantomography was normal (Fig. 1b). Measurement of bone mineral density (BMD) of the lumbar spine and the femoral neck after 1 year revealed osteoporosis below the expected range for age in a young adult (young adult mean [YAM], 87%; *T*-score, − 1.1; *Z*-score, − 1.1 in lumbar vertebra; YAM, 68%; *T*-score, − 2.5; *Z*-score, − 2.2 in femoral neck) (Table 2). Abdominal ultrasonography revealed numerous microcalcifications in both kidneys.

**Table 2:** Measurement of bone mineral density of lumbar spine and femoral neck

Variable	On presentation	After 1 year	After 2.5 years
T-score			
Lumbar vertebra	− 1.2	− 1.1	− 1.0
Femoral neck		− 2.5	− 2.5
Total hip		− 1.9	− 2.1
Z-score			
Lumbar vertebra	− 1.3	− 1.1	− 0.9
Femoral neck		− 2.2	− 2.2
Total hip		− 1.8	− 1.8
Young adult mean (age-matched) (%)			
Lumbar vertebra	86	87	89
Femoral neck		68	68
Total hip		76	75

On the basis of reduced BMD, additional blood chemistry tests and urinalysis were performed. The following bone metabolic markers were within normal limits: tartrate-resistant acid phosphatase-5b, 213 mU/dl (reference, 120–420 mU/dl); undercarboxylated osteocalcin, 2.14 ng/ml (reference, < 4.5 ng/ml); and type I procollagen N-terminal propeptide, 33.2 ng/ml (reference, 16.8–70.1 ng/ml). However, bone-specific ALP was low at 1.0 μg/L (reference, 2.9–14.5 μg/L) for her age. Analysis of amino acids in urine revealed that phosphoethanolamine was elevated at 727.8 μmol/g Cr (reference, 7–70 μmol/g Cr), which supported the diagnosis of HPP [10].

Serum ALP of the patient’s mother was low at 86 U/L. Therefore, the patient underwent genetic testing, which revealed two mutations in tissue-nonspecific *ALPL* (exon 9, c.979T>C [p.Phe327Leu] and exon 11, c.1559delT) (Fig. 1c). These genetic abnormalities, which were previously reported, were consistent with HPP [9, 11–13]. On the basis of the clinical presentation, laboratory and imaging findings, and genetic analyses, the patient was definitively diagnosed with adult HPP. She comes to our outpatient clinic every 6 months, and we have checked her BMD and abdominal ultrasonography every year for 3 years. Her medical conditions have been stable. Although we have not given her any medicine, we plan to introduce enzyme replacement therapy using human recombinant TNSALP when her disease state worsens.

## Discussion and conclusions

Our patient did not report any symptoms other than mild lower limb pain of unknown cause, and her low serum ALP value was found incidentally during an annual medical checkup for workers, which led to her referral to our hospital. Low serum ALP and pain in the lower limbs suggested a diagnosis of HPP, and analysis of amino acids in urine revealed elevation of phosphoethanolamine, which supported the diagnosis of HPP. Moreover, measurement of BMD at the femoral neck indicated osteopenia, whereas *ALPL* sequencing revealed two mutations, c.1559delT and c.979T>C (p.Phe327Leu), which are the most and second-most frequent mutation in Japanese patients, respectively [12, 13]. Interestingly, these compound heterozygous mutations, reported in perinatal benign, infantile, and childhood HPP types, were rarely reported in symptomatic patients with adult HPP [9]. On the basis of these results, the patient was diagnosed with adult HPP. Adult HPP typically presents in middle-aged patients and is diagnosed on the basis of symptoms, such as painful femoral pseudofractures, chronic muscle or bone pain, muscle weakness, pseudogout, and osteomalacia [6–8]. However, our patient had no history of childhood rickets or pseudofractures and had an abnormal serum level of ALP, which was incidentally found during an annual medical checkup. This was a unique reason for further referral and evaluation at our hospital. Therefore, the diagnosis of HPP was derived from an initial finding of a low serum level of ALP.

In our patient, the risk of bone fracture was high because of the low BMD below the expected range for her age, and she was osteopenic according to the International Society for Clinical Densitometry criteria. Because risk of death increases approximately twofold when associated with hip fractures due to osteoporosis, fracture prevention is critical [14]. Although bisphosphonates are usually used for the treatment of osteoporosis, some case studies have reported that treatment with bisphosphonates might potentially lead to an increase in and worsening of fractures [15–17]. Moreover, the human parathyroid hormone teriparatide is a controversial treatment option; although it was shown to provide some benefit in case studies of adults with HPP, one case report described no benefit [18–21]. As described later, most of the manifestations of adult HPP are atypical, and there might be many asymptomatic HPP “carriers” who have not been diagnosed. Some patients with adult HPP might be undiagnosed before they are initiated on bisphosphonates for postmenopausal osteoporosis. Because treatment approaches differ between osteoporosis and HPP, adult HPP should be considered in patients with low BMD.

The utility of disease-modifying treatment with enzyme replacement therapy using human recombinant TNSALP was reported recently [22–25]. Asfotase alfa (Strensiq™; Alexion Pharmaceuticals, New Haven, CT, USA) is a recombinant, bone-targeted, human TNSALP developed to treat the skeletal mineralization defects in HPP, and it reduces the accumulation of extracellular TNSLP substrates. The goal of the enzyme replacement treatment in patients with adult HPP without fractures is improvement in functional status as measured by strength, endurance, improvement in gait, reduction in fatigue, and prevention of fractures, all of which improve mobility and quality of life. However, questions regarding the quality of life and long-term sustainability of this high-cost treatment cannot yet be answered [26]. Our patient had no history of fractures, tooth abnormalities, or skeletal dysfunction. Although the risk of fracture remained, we decided to follow the patient without initiating enzyme replacement therapy. Further studies on enzyme replacement therapy in patients with adult HPP are needed.

Our patient carried compound heterozygous missense and frameshift mutations in *ALPL*. Some patients with perinatal HPP are homozygous, with asymptomatic parents who are heterozygous carriers for the mutations. p.Phe327Leu and c.1559delT are more common in Japanese patients with HPP [9, 11], whereas p.Glu191Lys and p.Asp378Val occur more frequently in Caucasian patients [27]. In our patient, genetic testing revealed c.1559delT homozygous mutation and c.979T>C (p.Phe327Leu) in *ALPL*, which are associated with lethal and the perinatal nonlethal (benign) forms of HPP in Japanese patients, respectively. One reason underlying this difference in HPP phenotypes is proposed to be due to differences in enzymatic activity associated with the mutations. The enzymatic activity of ALP is 70% with p.Phe327Leu, whereas its activity is completely abolished with c.1559delT. However, in the majority of carriers with the c.1559delT mutation, biomarkers, serum ALP activity, and urinary phosphoethanolamine levels are within normal limits [13]. Given the low level of serum ALP and high level of urinary phosphoethanolamine in our patient, it was interesting that the two heterozygous mutations caused the abnormal serum, urinary, and BMD parameters. Moreover, the compound heterozygous mutations of p.Phe327Leu and c.1559delT in *ALPL*, which were reported in perinatal benign, infantile, and childhood HPP types and carrier parents, were rarely reported in symptomatic adult patients with HPP [9]. Further investigation is needed to explore the mechanisms underlying the differential effects on these mutations.

Although adult HPP is considered to be the rarest phenotype, several studies reported cases of adult HPP [4, 7]. Because the clinical presentation of adult HPP is variable, including bone and muscle pain, joint problems, bone fractures, dental problems, and muscular insufficiency, morbidity of adult HPP is not well understood. Moreover, hypercalcemia, hyperphosphatemia, and kidney calcification are sometimes noted in adult HPP. Because the carrier frequency for c.1559delT is estimated to be 1 in 480 in the Japanese population [13], a considerable number of patients, who are predicted not to be recognized as carriers for HPP, might present with bone weakness attributable to osteoporosis. It is important to increase awareness regarding HPP for its appropriate treatment.

With the widely conducted routine health checkups in Japan, there are numerous opportunities to measure serum ALP levels during medical checkups for workers and residents. Because the underlying causes of low serum ALP levels are limited to steroid treatment, hypothyroidism, and zinc deficiency, HPP should be considered in the differential diagnosis of low serum ALP. Correct diagnosis of HPP is critical for prevention of bone fractures, reduction of pain, and improvement of quality of life.

## Abbreviations

ALP: Alkaline phosphatase
BMD: Bone mineral density
HPP: Hypophosphatasia
TNSALP: Tissue-nonspecific alkaline phosphatase
YAM: Young adult mean

## References

[1] Rathbun JC. Hypophosphatasia, a new developmental anomaly. Am J Dis Child. 1948;75:822–31. 10.1001/archpedi.1948.02030020840003 18110134

[2] Whyte MP. Hypophosphatasia. In: Thakker RV, Whyte MP, Eisman JA, Igarashi T, editors. Genetics of bone biology and skeletal disease. London: Academic Press; 2013. p. 337–60.

[3] Mornet E. The tissue nonspecific alkaline phosphatase gene mutations database. http://www.sesep.uvsq.fr/03_hypo_mutations.php. Accessed 6 Sept 2018.

[4] Whyte MP, Teitelbaum SL, Murphy WA, Bergfeld MA, Avioli LV. Adult hypophosphatasia: clinical, laboratory, and genetic investigation of a large kindred with review of the literature. Medicine (Baltimore). 1979;58:329–47. 481194

[5] Berkseth KE, Tebben PJ, Drake MT, Hefferan TE, Jewison DE, Wermers RA. Clinical spectrum of hypophosphatasia diagnosed in adults. Bone. 2013;54:21–7. 10.1016/j.bone.2013.01.024 23352924

[6] Weber TJ, Sawyer EK, Moseley S, Odrljin T, Kishnani PS. Burden of disease in adult patients with hypophosphatasia: results from two patient-reported surveys. Metabolism. 2016;65:1522–30. 10.1016/j.metabol.2016.07.006 27621187

[7] Conti F, Ciullini L, Pugliese G. Hypophosphatasia: clinical manifestation and burden of disease in adult patients. Clin Cases Miner Bone Metab. 2017;14:230–4. 10.11138/ccmbm/2017.14.1.230 PMC5726215 29263739

[8] McKiernan FE, Berg RL, Fuehrer J. Clinical and radiographic findings in adults with persistent hypophosphatasemia. J Bone Miner Res. 2014;29:1651–60. 10.1002/jbmr.2178 24443354

[9] Taketani T, Onigata K, Kobayashi H, Mushimoto Y, Fukuda S, Yamaguchi S. Clinical and genetic aspects of hypophosphatasia in Japanese patients. Arch Dis Child. 2014;99:211–5. 10.1136/archdischild-2013-305037 24276437

[10] Rasmussen K. Phosphorylethanolamine and hypophosphatasia. Dan Med Bull. 1968;15(Suppl 2):1–112. 4306219

[11] Ozono K, Yamagata M, Michigami T, *et al.* Identification of novel missense mutations (Phe310Leu and Gly439Arg) in a neonatal case of hypophosphatasia. J Clin Endocrinol Metab. 1996;81:4458–61. 10.1210/jcem.81.12.8954059 8954059

[12] Michigami T, Uchihashi T, Suzuki A, Tachikawa K, Nakajima S, Ozono K. Common mutations F310L and T1559del in the tissue-nonspecific alkaline phosphatase gene are related to distinct phenotypes in Japanese patients with hypophosphatasia. Eur J Pediatr. 2005;164:277–82. 10.1007/s00431-004-1612-9 15660230

[13] Watanabe A, Karasugi T, Sawai H, *et al.* Prevalence of c.1559delT in ALPL, a common mutation resulting in the perinatal (lethal) form of hypophosphatasia in Japanese and effects of the mutation on heterozygous carriers. J Hum Genet. 2011;56:166–8. 10.1038/jhg.2010.161 21179104

[14] LeBlanc ES, Hillier TA, Pedula KL, *et al.* Hip fracture and increased short-term but not long-term mortality in healthy older women. Arch Intern Med. 2011;171:1831–7. 10.1001/archinternmed.2011.447 PMC3576923 21949033

[15] Whyte MP. Atypical femoral fractures, bisphosphonates, and adult hypophosphatasia. J Bone Miner Res. 2009;24:1132–4. 10.1359/jbmr.081253 19113923

[16] Sutton RA, Mumm S, Coburn SP, *et al.* “Atypical femoral fractures” during bisphosphonate exposure in adult hypophosphatasia. J Bone Miner Res. 2012;27:987–94. 10.1002/jbmr.1565 22322541

[17] Cundy T, Michigami T, Tachikawa K, *et al.* Reversible deterioration in hypophosphatasia caused by renal failure with bisphosphonate treatment. J Bone Miner Res. 2015;30:1726–37. 10.1002/jbmr.2495 25736332

[18] Camacho PM, Mazhari AM, Wilczynski C, Kadanoff R, Mumm W, Whyte MP. Adult hypophosphatasia treated with teriparatide: report of 2 patients and review of the literature. Endocr Pract. 2016;22:941–50. 10.4158/EP15890.OR 27042741

[19] Whyte MP, Mumm S, Deal C. Adult hypophosphatasia treated with teriparatide. J Clin Endocrinol Metab. 2007;92:1203–8. 10.1210/jc.2006-1902 17213282

[20] Righetti M, Wach J, Desmarchelier R, Coury F. Teriparatide treatment in an adult patient with hypophosphatasia exposed to bisphosphonate and revealed by bilateral atypical fractures. Joint Bone Spine. 2018;85:365–7. 10.1016/j.jbspin.2017.12.001 29246529

[21] Laroche M. Failure of teriparatide in treatment of bone complications of adult hypophosphatasia. Calcif Tissue Int. 2012;90:250. 10.1007/s00223-011-9562-5 22218563

[22] Whyte MP, Greenberg CR, Salman NJ, *et al.* Enzyme replacement therapy in life-threatening hypophosphatasia. N Engl J Med. 2012;366:904–13. 10.1056/NEJMoa1106173 22397652

[23] Whyte MP, Rockman-Greenberg C, Ozono K, *et al.* Asfotase alfa treatment improves survival for perinatal and infantile hypophosphatasia. J Clin Endcrinol Metab. 2016;101:334–42. 10.1210/jc.2015-3462 PMC4701846 26529632

[24] Whyte MP, Madson KL, Phillips D, *et al.* Asfotase alfa therapy for children with hypophosphatasia. JCI Insight. 2016;1(9):e85971. 10.1172/jci.insight.85971 PMC5033855 27699270

[25] Kitaoka T, Tajima T, Nagasaki K, *et al.* Safety and efficacy of treatment with asfotase alfa in patients with hypophosphatasia: results from a Japanese clinical trial. Clin Endocrinol. 2017;87:10–9. 10.1111/cen.13343 28374482

[26] Costain G, Moore AM, Munroe L, *et al.* Enzyme replacement therapy in perinatal hypophosphatasia: case report of a negative outcome and lessons for clinical practice. Mol Genet Metab Rep. 2018;14:22–6. 10.1016/j.ymgmr.2017.10.006 PMC5681336 29159075

[27] Mornet E. Hypophosphatasia. Best Pract Res Clin Rheumatol. 2008;22:113–27. 10.1016/j.berh.2007.11.003 18328985

